# Anxiety and depressive features in chronic disease patients in Cambodia, Myanmar and Vietnam

**DOI:** 10.4102/sajpsychiatry.v22i1.940

**Published:** 2016-07-28

**Authors:** Karl Peltzer, Supa Pengpid

**Affiliations:** 1 ASEAN Institute for Health Development, Madidol University, Thailand; 2 Department of Research Innovation and Development, University of Limpopo, South Africa; 3 HIV/AIDS/STIs and TB (HAST), Human Sciences Research Council, Pretoria, South Africa

## Abstract

**Objective:**

The aim of this study was to estimate the prevalence and relationship of anxiety and depressive features among patients diagnosed with a variety of chronic diseases in three Southeast Asian countries (Cambodia, Myanmar and Vietnam).

**Methods:**

A cross-sectional survey was conducted in 2014 among 4803 adult patients with chronic diseases who were recruited cross-sectionally from health facilities. Anxiety and depression were assessed with the *Hospital Anxiety and Depression Scale*.

**Results:**

Overall, 17.0% of patients screened positive for anxiety disorder and 39.1% for depressive disorder. Patients with cancer (47.8%) had the highest rate of anxiety features, and those with chronic obstructive pulmonary disease (COPD) (62.1%), kidney disease (55.5%), Parkinson’s disease (53.7%) and cardiovascular disorders (CVDs) (52.6%) the highest prevalence of depressive features. Stomach and intestinal diseases, CVDs, migraine or frequent headaches and kidney disease were positively associated with anxiety and depression after adjusting for sociodemographics and illness duration. In addition, cancer and Parkinson’s disease were positively associated with anxiety, and arthritis, diabetes, and COPD were positively associated with depression. In multivariate logistic regression, having two or more chronic conditions and poor quality of life was associated with anxiety and depression.

**Conclusion:**

Considering the high rate of anxiety and depression among these patients with chronic disease, more efforts should directed to on the psychosocial management of these patients.

## Introduction

Depression and anxiety are common in people with chronic diseases.^1^ Clark and Currie^2^ (p. 190) reviewed that ’depression is more common in patients with heart disease, stroke, diabetes mellitus, asthma, cancer, arthritis and osteoporosis and anxiety is more common in people with heart disease, stroke and cancer than in the general population’. Cross-sectional studies have reported that the prevalence of co-morbid anxiety and depression is high in patients with chronic heart failure and chronic obstructive pulmonary disease (COPD).^3^ Scott et al.^4^ found that anxiety and depression were associated with diabetes, asthma, hypertension, arthritis, ulcer, heart disease, back/neck problems, chronic headache and multiple pains. Anxiety disorders were associated with hypertension.^5^ Hochstrasser and Angst^6^ found that stomach and intestinal complaints were associated with depressive and anxiety disorders. Psychiatric co-morbidities, particularly mood and anxiety disorders, were found to be more common among persons with migraine or frequent headaches.^7^ In Latinos living in the United States, anxiety was associated with diabetes and cardiovascular disease and depression and co-occurring anxiety and depression were positively associated with having a history of asthma.^8^ Anxiety disorders were also found to be associated with Parkinson’s disease^9^ and anxiety and depressive disorders with kidney disease.^10^

Katon, Lin and Kroenke^11^ (p. 153) found that patients with chronic medical illness and co-morbid depression or anxiety compared to those with chronic medical illness alone reported significantly higher numbers of medical symptoms when controlling for severity of medical disorder. Similarly, a higher number of co-morbidities was associated with anxiety and depression.^12^

In studies in low- and middle-income countries, for example, Turkey, it was found that the prevalence of anxiety disorder in hypertension was 9.5% and that for diabetes was 16.0%^13^ and a prevalence of depressive disorder in hypertension was 5.2% and that for diabetes was 22.7%.^13^ In patients with diabetes, co-morbid depression was found to have a prevalence of 23.0% – 39.2% in China and 16.9% – 84.0% in India.^14^ The prevalence of depression was 28.3% in patients with chronic disease (arthritis 10.6%, diabetes 48.1% and heart disease 20.2%) in Trinidad.^15^ Among Indian patients with chronic disease, 14.7% mental distress was found in patients with diabetes, 17.1% in those with hypertension and 13.3% in those with arthritis.^12^ In a population undergoing haemodialysis in Nigeria, 35.0% had a major depressive episode and 29.0% had a generalised anxiety disorder.^10^

In systematic reviews, the prevalence of depression using self- or clinician-administered rating scales in patients with chronic kidney disease was found to be 39.3%,^16^ and in a review, the prevalence of depressive disorder was found to be 14.8% in patients with arthritis.^17^ Furthermore, anxiety and depressive disorders in patients with chronic diseases have been found to be associated with poorer quality of life^18^ and various sociodemographic factors such as low educational level.^15^

The aim of this study was to estimate the prevalence and relationship of anxiety and depressive features among patients diagnosed with a variety of chronic diseases in three Southeast Asian countries (Cambodia, Myanmar and Vietnam).

## Methods

### Sample and procedure

In each Southeast Asian country (Cambodia, Myanmar, Vietnam), a cross-sectional survey in conveniently sampled rural and urban health facilities was conducted on outpatients with chronic diseases. The sample size included at least 800 persons from rural health facilities and 800 individuals from urban health facilities in each country. Every eligible patient (18 years and above and having been treated for a chronic disease in the past 12 months)^19^ was selected from the health facility, using a consecutive sampling procedure. Health facility staff identified patients with chronic diseases using medical file information and referred eligible patients to interviewers. Trained research assistants conducted interviews with the patients using structured questionnaires after informed consent was obtained. In Cambodia, the National Ethics Committee for Health Research of Ministry of Health in Cambodia (Reference no: 0225NECHR), in Myanmar the Research and Ethical Committee of University of Medicine 1, Yangon, in Vietnam The Committee of Research Ethics of Hanoi School of Public Health, and in Thailand, The Committee of Research Ethics (Social Sciences) of Mahidol University (COA.No.: 2014/193.0807) approved the study protocol.

### Measures

The Hospital Anxiety and Depression Scale (HADS)^20^ was used to assess anxiety (7 items) and depression (7 items). Scores of 11 or more were used as cut-offs for anxiety and depression, respectively, as recommended by Zigmond and Snaith.^20^ HADS subscales were internally reliable (α anxiety: 0.73; α depression: 0.71).

Sociodemographic information included age, gender, country, formal education, marital status, employment status and residency status.

Quality of life was assessed by the World Health Organization Quality of Life (WHOQol)-8 consisting of eight items that were derived from the WHOQOL-Bref.^21^ The summative model was used in this study to generate an index. Cronbach’s alpha for the WHOQol-8 was 0.89 in this sample.

### Data analysis

The data were analysed using IBM SPSS (version 20.0). Associations between key outcomes of anxiety and depression and specific chronic conditions were evaluated calculating odds ratios (ORs), while adjusting for age, gender, education, geolocality and duration of chronic condition. Unconditional multivariable logistic regression was used for the evaluation of the impact of explanatory variables for the key outcomes of anxiety and depression (binary dependent variables), *p* < 0.05 was considered significant.

## Results

### Sample characteristics and prevalence rate of mental morbidity

The total sample included 4803 adults, 1602 in Cambodia, 1600 in Myanmar and 1601 in Vietnam, and the mean age was 49.3 years (standard deviation = 16.5; range, 18–94 years); 69.6% were female participants. More than half of the participants (59.0%) were 46 years or older, and 55.4% had been diagnosed with at least one chronic disease in the past 12 months. The educational level of most participants (77.8%) was lower than secondary school education, and 55.4% lived in rural areas (see Table 1).

**TABLE 1:** Sample characteristics and prevalence of mental morbidity.

Chronic conditions	*N* (%)	Anxiety	Depression
		*N* (%)	*N* (%)
All	4803 (100)	803 (17.0)	1843 (39.1)
Stomach and intestinal diseases	1935 (40.3)	374 (19.7)	934 (49.7)
Hypertension	1402 (29.2)	215 (15.6)	623 (45.0)
Arthritis	909 (18.9)	171 (19.6)	440 (51.0)
Cardiovascular disorders (cardiac failure, coronary artery disease, cardiac arrhythmias, stroke)	804 (16.8)	172 (22.0)	409 (52.6)
Gout and other musculoskeletal conditions, such as chronic backache	748 (15.6)	119 (16.4)	219 (29.9)
Diabetes mellitus	509 (10.6)	94 (18.5)	249 (49.1)
Migraine or frequent headaches	350 (7.3)	79 (23.7)	168 (49.6)
Chronic obstructive pulmonary disease	308 (6.4)	54 (17.8)	185 (62.1)
Kidney disease	240 (5.0)	63 (27.4)	127 (55.5)
Liver disease	234 (4.9)	32 (13.9)	53 (23.0)
Asthma	219 (4.6)	30 (14.0)	69 (31.8)
Dyslipidaemia	210 (4.4)	30 (14.8)	63 (30.9)
Thyroid disease	75 (1.6)	10 (13.7)	20 (27.0)
Cancer	72 (1.5)	33 (47.8)	23 (33.3)
Parkinson’s disease	69 (1.4)	24 (35.3)	36 (53.7)

In terms of mental morbidity among patients with chronic disease, 17.0% screened positive for anxiety disorder and 39.1% for depressive disorder. Participants had been diagnosed for at least one chronic condition in the past 12 months, of which the six major ones were stomach and intestinal diseases (40.3%), hypertension (29.2%), cardiovascular disease (16.8%), arthritis (18.9%), gout and other musculoskeletal conditions, such as chronic backache (15.6%), and diabetes (10.6%). Among the chronic disease conditions, patients with cancer (47.8%) had the highest rate of anxiety features. Depressive features were the highest among patients with COPD (62.1%), followed by kidney disease (55.5%), Parkinson’s disease (53.7%) and cardiovascular disorder (CVD) (52.6%) (see Table 1).

### Associations for chronic conditions with anxiety and depression

In multivariate logistic regression analyses adjusted for age, gender, education, geolocality and duration of chronic condition, stomach and intestinal diseases (OR = 1.25, CI = 1.06–1.47), CVDs (OR = 1.36, CI = 1.12–1.66), migraine or frequent headaches (OR = 1.42, CI = 1.08–1.88), kidney disease (OR = 1.85, CI = 1.36–2.53), cancer (OR = 5.17, CI = 3.12–8.56) and Parkinson’s disease (OR = 2.15, CI = 1.26–3.69) were positively associated with anxiety, and hypertension (OR = 0.77, CI = 0.63–0.92) was negatively associated with anxiety. Furthermore, stomach and intestinal diseases (OR = 2.22, CI = 1.92–2.55), arthritis (OR = 1.62, CI = 1.37–1.92), CVDs (OR = 1.63, CI = 1.38–1.94), diabetes (OR = 1.41, CI = 1.14–1.73), migraine or frequent headaches (OR = 1.44, CI = 1.13–1.85), COPD (OR = 1.78, CI = 1.38–2.30) and kidney disease (OR = 1.67, CI = 1.26–2.21) were positively associated with depression, while gout and other musculoskeletal conditions (OR = 0.67, CI = 0.55–0.80), and asthma (OR = 0.61, CI = 0.44–0.83) were negatively associated with depression.

### Associations with anxiety and depression

In multivariate logistic regression, being of female sex (OR = 0.76, CI = 0.66–0.87), full-time employed (OR = 1.60, CI = 1.37–1.86), having three or more chronic conditions (OR = 1.66, CI = 1.39–1.98) and poor quality of life (OR = 0.53, CI = 0.46–0.60) were associated with anxiety, while older age (OR = 1.48, CI = 1.22–1.81), lower education (OR = 0.16, CI = 0.13–0.20), rural residence (OR = 0.54, CI = 0.47–0.62) having three or more chronic conditions (OR = 2.92, CI = 2.41–3.53) and poor quality of life (OR = 0.53, CI = 0.45–0.62) were associated with depression.

## Discussion

Similar to the results obtained in previous studies,^2,3,4,6,7,9,10^ this study also found that stomach and intestinal diseases, CVDs, migraine or frequent headaches, kidney disease, cancer and Parkinson’s disease were positively associated with anxiety. Unlike previous studies,^4,5,8^ this study did not find an association between asthma, hypertension and anxiety.

Furthermore, in agreement with previous studies,^2,3,4,6,7,10^ this study found that stomach and intestinal diseases, arthritis, CVDs, diabetes, migraine or frequent headaches, COPD and kidney disease were positively associated with depression. In contrast to previous studies,^2,4,8^ this study found that gout and other musculoskeletal conditions and asthma were negatively associated with depression. It may need to be considered that in this study sample only patients with chronic disease were included, and they were not compared with the general population, which may have depleted in some cases the impact of anxiety and depressive features on some specific conditions such as asthma and hypertension.

Overall, a high prevalence of anxiety (17.0%) and depressive features (39.1%) were found in this study among patients with chronic diseases in Southeast Asian countries, which seemed to be higher than in a previous study among patients with chronic diseases in Trinidad.^15^ Compared to other world regions, Mendenhall et al.^14^ confirmed a high prevalence of depression in patients with diabetes in East and South Asia. The prevalence of anxiety and depressive features in the various chronic conditions was largely similar or higher than what was found in previous studies, for example, studies regarding co-morbid depression in diabetes patients,^14,15^ co-morbid hypertension,^13^ co-morbid CVD,^15^ co-morbid arthritis^15^ and co-morbid kidney disease.^10^ Some of the comparisons across studies may be difficult because different assessment measures such as screens or psychiatric interviews were used. Nevertheless, the study findings call for better attention to identify and manage mental morbidity in patients with chronic diseases in this region.^22^

Furthermore, the study found that with an increase in the number of chronic conditions, patients were more likely to screen positive for anxiety and depression features. This finding confirms previous studies.^12,15^ Some biological theories talk about the interactions between anxiety and specific chronic diseases such as CVDs and chronic pain.^22^ This information about the association of anxiety and depressive features with multimorbidity and multiple chronic conditions may help in the clinical management.^15^ As also found previously,^18^ this study observed that anxiety and depressive features were associated with poorer quality of life. This finding further complicates the management of patients with co-morbid chronic disease. As also found in earlier studies,^15^ some sociodemographic factors (such as low education) were found to be associated with depression in patients with chronic disease. It may be that those who are more educated have more resources to cope with life and their chronic disease.^15^

## Study limitations

There were several study limitations. The study was cross-sectional in nature, and no causal conclusions can be drawn. Furthermore, the sample size of some of the physical chronic conditions was small, which limits the interpretation of these results. Also, there was a lack of a comparison group of patients with chronic conditions. In order to investigate the direction of the relationship between mental morbidity and physical chronic conditions, more longitudinal studies are needed.

## Conclusion

The study found a high prevalence of anxiety and depression among patients with chronic diseases. Both anxiety and depression should not be ignored and could be considered conjointly when developing strategies for screening and management of patients with chronic disease. Several specific chronic conditions, including cancer, COPD, kidney disease, Parkinson’s disease, CVDs and multiple chronic conditions, were identified to be particularly vulnerable to anxiety and depressive features and may be more specifically targeted in intervention programmes.
